# Variations of motility and survival with storage time at 4°C of epididymal spermatozoa Ouled-Djellal breed rams in Eastern Algeria

**DOI:** 10.14202/vetworld.2015.326-329

**Published:** 2015-03-16

**Authors:** B. Safsaf, S. Belkadi, L. Belkacem, B. Mamache, M. Tlidjane

**Affiliations:** Department of Veterinary Science, Laboratory ESPA, Veterinary and Agricultural Sciences Institute, Hadj Lakhdar University, Batna - 05000, Algeria

**Keywords:** breed ouled-djellal, epididymal sperm, rams, storage at 4°C

## Abstract

**Aim:**

The aim of this study was to evaluate some reproduction performances in Ouled-Djellal rams.

**Materials and Methods:**

This study involved genital organs removed after slaughter from 54 rams at the municipal slaughterhouse of Batna (East Algeria).

**Results:**

The measurements of survival and mobility of epididymal sperm followed at 0, 24, 48 and 72 h after collection, revealed significant (p<0.05) to highly significant differences (p>0.001) according to time. Thus, concerning the sperm motility the values were 91.00±2.40%, 89.20±2.40%, 77.00±6.20% and 62.60±1.20% at 0, 24, 48 and 72 h, respectively. Indeed, in live sperm, the viability rates were 82.15±1.48%, 77.67±1.74%, 66.56±1.95% and 52.30±1.46% at 0, 24, 48 and 72 h, respectively.

**Conclusion:**

This study revealed that epididymal spermatozoa stored at 04°C for 72 h kept their mobility and vitality at nearly a half of their the original parameters.

## Introduction

In Algeria, sheep raising is concentrated in the steppe and the mutton is the most favorable meat. Ouled-Djellal (OD) breed is the most dominant in this region representing nearly 60% of the 22.868 million heads [1]. This is an all-white sheep. For its best qualities, this breed tends to dominate other blood and improving its fertility to increase livestock productivity and reproductive efficiency. This goal can be achieved by improving the reproductive performances of the rams. These must have superior reproductive trait to improve the genetic merit of a flock and to reduce the number of breeding males to increase flock fertility [2]. Reproductive capacity of rams is directly or indirectly involved in the reproductive process, either during natural reproduction or by the production of semen for artificial insemination. The immature spermatozoa produced in the testis over a period of several days will be released in the epididymis. The recourse to post-mortem recovery of epididymal sperm could be useful, in the event that a genetically interesting male dies accidentally or must be culled (disease-carriers, physical defects, illness, etc.) [3] and also helps greatly to preserve biodiversity [4-6]. The preservation of genetic material from several species has been passed on using sperm extracted from the epididymis of the testis. This process has been described in the stallion [7], dog [8], African buffalo [9], bull [10], brown bear [11], goat [12,13], ram [4,14], red deer [5] and Spanish ibex [15]. The collection of epididymal sperm allows the collection of spermatozoa in sufficient numbers for artificial inseminations [16]. However, in order to get good quality samples, sperm collection and processing should be carried out immediately after the death of the animal. This is not always possible, especially regarding wild species, but it is possible in domestic ones. Even though, sperm cells can survive for some time in the epididymis of dead animals. Their quality deteriorates with time, because of the changes related to body death and decomposition [8,17,18]. According to Varisli *et al.*, [19], epididymal ram sperm is extremely resistant to various cryobiologically active stress conditions than ejaculated sperm that demonstrates greater sensitivity to stressors like chilling.

The aim of the present study was to determine the effect of the storage time on the quality of epididymal spermatozoa kept at 4°C by evaluating their motility and viability at 0, 24, 48, and 72 h after the rams’ slaughter.

## Materials and Methods

### Ethical approval

Ethical approval was not necessary. The samples were taken from slaughtered animals.

### Epididymal sperm

This study involved 54 OD rams at the municipal slaughterhouse of Batna (East Algeria). The genital organs were removed after slaughter of the animal. Before the collection of sperm, a paraffin oil injection was performed into the vas deferens, after which an incision in the apical region of the tail was carried out, and the epididymal sperm was collected and stored at 4°C without dilution [16]. Mobility and vitality were measured at 0, 24,48 and 72 h after semen collection. A drop of semen was observed by light microscopy at low magnification (×10) to assess the massal motility graded from 0 to 5 (0=no movement, 5=Massal motility with vortex). However, the vitality was assessed by counting after Eosin-nigrosin staining, to determine the percentage of live (eosin-negative) spermatozoa [20].

### Statistical analysis

We used the Software Graph Pad Prism^®^5. Version 5.03, to calculate the mean, the standard deviation and the standard error of the mean (SEM) and the statistical signification was set at p<0.05.

## Results

### Motility and viability of cauda epididymal spermatozoa

The analysis of massal motility and vitality are summarized in Table-1. The data revealed that massal motilty of cauda epididymal spermatozoa tended to diminish with the evolution of time and varied from 4.55±0.12 (0 h) to 3.133±0.06 (72 h). The results showed a significant difference (p<0.05) among times of storage. The vitality rate showed a decrease with elapsing time and varied from 82.15±1.48% at 0 h to 52.30±1.46% at 72 h. Highly significant differences (p<0.001) were observed between the periods 0 h versus 48 h 0 h versus 72 h, 24 versus 48, 24 versus 72 h and 48 h versus 72 h.

**Table-1:** Variations at times (0, 24, 48 and 72 hr) of massal motility (0-5) and viability rate (%) of epididymal spermatozoa stored at 4°C.

Time of storage (h)	0	24	48	72	Statistical significance
Massal motility (0-5)	4.55±0.12	4.46±0.12	3.85±0.31	3.13±0.06	^abcdef^*
%	91.00±2.40	89.20±2.40	77.00±6.20	62.60±1.20	
Viability of spermatozoa (%)	82.15±1.48	77.67±1.74	66.56±1.95	52.30±1.46	^bcdef^***

a 0 h versus 24 h;

b 0 h versus 48 h;

c 0 h versus 78 h;

d 24 h versus 48 h;

e 24 h versus72 h and

f 48 h versus 72 h,

* p<0.05;

*** p<0.001

## Discussion

The post-mortem recovery of epididymal sperm from dead animals is a useful method that would permit the creation of germplasm banks to preserve endangered breeds and contributes to the preservation of biodiversity [4]. The sperm undergoes two types of motility, progressive motility, and hyperactivated motility. They attain the ability for progressive motility during epididymal maturation but do not become motile until released from the epididymis [21]. With storage in the cauda epididymis, a loss in fertilizing ability was found to occur before a loss in motility [22]. Motility of spermatozoa held in the epididymal fluid and stored either in the cauda epididymis, after 24 h post-mortem, was higher compared to those held *in vitro* with extended media, although differences decreased at 48 and 72 h [6]. In bulls, Martins *et al.*, [23] obtained a decline in total motility after 48 h of storage, which remained stable until 72 h. Sperm mobility was slightly higher to that obtained by Lones *et al.*, [24] and Mir *et al.*, [25]. Lones *et al.*, [24] have obtained an overall sperm motility after refrigeration (4.9-6°C) and ambiant (17.9-21.5°C) temperatures of 82.50% at 0 h and 75.00%, at 24 h, 73.33% and 67.50%, at 48 h was 67.50% and 55.83%, respectively, and the corresponding values at 72 h of preservation were 60.00% and 45.83%. In the same case, Mir *et al.*, [25] have noted a decline of almost half at 48 h in progressive motility with 82.50% and 83.3% at 0 h to 67.50% and 44.17% at 48 hr, respectively. In live sperm, our values were lower than those obtained by Lones *et al.*, [24] and Mir *et al.*, [25] in rams, and by Martins *et* *al*., [23] in bulls with 64.00±15.4% after 72 h of storage at 5°C. The two first ones have obtained in mean sperm viability at 0 h for refrigeration and ambient temperature 92.92% and 88.92%, respectively, and the corresponding records at 72 h, 81.50%, and 73.17%. As for the second ones, they got slightly less values with 93.85±1.98 at 0 h to 78.91±1.77 at 48 h.

The motility of spermatozoa varies with the transport temperature (ambient or refrigeration) [24] and its origin [6]. These authors, noted a significant effect on the progressive motility and kinematic parameters according to the method of collection of the sample (ejaculation, epididymal or electroejaculation) and the media added during pre-freezing; and they found that epididymal spermatozoa had the greatest values for curvilinear velocity and viability, and the lowest pourcentage of damaged acrosomes. It has also been observed a variation of the quality of the semen (progressive motility, vitality, and plasma membrane functional integrity) during the pre-freezing according to the cryoprotective agents. In goats, Blash *et al.*, [12] did not find significant differences between epididymal and ejaculated sperms, in parameters such as progressive motility, viability, and membrane integrity, neither before nor after freezing. In stallion, Braun *et* *al*., [7] have noted in epididymal and ejaculated spermatozoa storedat 5°C, that the relative loss of motility was less pronounced in epididymal than in ejaculated spermatozoa. In the same case, Rath, and Niemann, [26] obtained in pig high *in vitro* fertilization (IVF) rates of *in vivo* matured oocytes with epididymal sperm, and conceived that epididymal sperm was better for IVF than ejaculated semen due to deficiency of contact with seminal plasma.

According to Tamayo-Canul *et al.*, [14], the storage of the spermatozoa of the ram in the epididymis is a good approach for maintaining its quality for at least 48 h. Thus, Kaabi *et al.*, [4] revealed that epididymal sperm stored at 5°C showed a better motility and a lower percentage of abnormal forms than epididymal sperm stored at room temperature during 24, and 48 h. Mature cauda epididymal sperm is a better model than ejaculated sperm because the latter is exposed to a variety of undefined constituents from the secretions of seminal vesicles and prostate [13]. However, when semen is used in artificial insemination, the addition of seminal plasma with epididymal spermatozoids permits to improve their capacity to cross the cervix compared to those used without seminal plasma at the time of insemination. It has been confirmed by the rates and the number of spermatozoids observed at the utero-tubal junction, and the number of gestations was meaningfully more elevated compared to the epididymal spermatozoids not exposed to seminal plasma [27]. It is well established that epididymal sperm motility is acutely affected by time of storage in different species [4,8,18,28]. Some motility parameters are altered as soon in a few hours post mortem, and that these motility changes followed by other characteristics, such as morphology or viability. The loss of energy during postmortem could be responsible for the loss of maintenance of the membrane with a consequent increase in permeability leading to a decrease in sperm viability and motility, losing linearity and speed [15]. In Iberian red deer and roe deer, the percentages of motile and progressive spermatozoa dropped just after the first 24 h post-mortem without significant differences before 48 h [18]. Finally, Mir *et* *al*., [25] concluded that ram epididymis could be stored at 4°C for 48 h when epididymal spermatozoa cannot be immediately collected and cryopreserved.

## Conclusion

This study showed that epididymal sperm can be used for more or less short time after storage. A storage time up to 72 h at 4°C can lead to a reduction of nearly half in motility and viability rates. We can conclude that the cauda epididymal sperm stored at the above-mentioned conditions constitutes, despite an obvious reduction in viability, an alternative source of gametes of meritorious parents for artificial insemination or IVF. However, for a better evaluation of the fertility or performance, rams should be tested for different trials such as scrotal measurement, semen examination, libido testing, hormonal profile and other examinations.

## References

[1] MADR (Ministry of Agriculture and Rural Development- Algeria). Algeria Report (1). Current situation of breeding camels in arid and semi-arid areas International Workshop on: The impact of climate change on livestock and sustainable rangeland management in arid and semi-arid areas of North Africa.

[2] Mukasa-Mugerwa E, Ezaz Z (1992). Relationship of testicular growth and size to age, body weight and onset of puberty in Menzram lambs. Theriogenology.

[3] Ehling C, Rath D, Struckmann C, Frenzel A, Schindler L, Niemann H (2006). Utilization of frozen-thawed epididymal ram semen to preserve genetic diversity in Scrapie susceptible sheep breeds. Theriogenology.

[4] Kaabi M, Paz P, Alvarez M, Anel E, Boixo JC, Rouissi H, Herraez P, Anel L (2003). Effect of epididymis handling conditions on the quality of ram spermatozoa recovered post-mortem. Theriogenology.

[5] Martinez-Pastor F, Diaz-Corujo AR, Anel E, Herraez P, Anel L, de Paz P (2005). Post mortem time and season alter subpopulation characteristics of Iberian red deer epididymal sperm. Theriogenology.

[6] Álvarez M, Tamayo-Canul J, Martínez-Rodrígueza C, López-Uruena E, Gomes-Alves S, Anel L, Martínez-Pastor F, de Paz P (2012). Specificity of the extender used for freezing ram sperm depends of the spermatozoa source (ejaculate, electroejaculate or epididymis). Anim. Reprod. Sci.

[7] Braun J, Sakai M, Hochi S, Oguri N (1994). Preservation of ejaculated and epididymal stallion spermatozoa by cooling and freezing. Theriogenology.

[8] Songsasen N, Tong J, Leibo S (1998). Birth of live mice derived by *in vitro* fertilization with spermatozoa retrieved up to twenty-four hours after death. J. Exp. Zool.

[9] Lambrechts H, van Niekerk F.E, Coetzer W.A, Cloete S.W.P, van der Horst G (1999). The effect of cryopreservation on the survivability, viability and motility of epididymal African buffalo (Svnceruscaffer) spermatozoa. Theriogenology.

[10] Nichi M, Rijsselare T, Van Soom A, De Clercq J.B.P, Goovaerts I.G.F, Barnabe V.H, Bols P.E.J (2007). Effect of bull epididymis storage conditions on cryopreserved epididymal sperm *in vitro* fertility and lipid peroxidation status. 33rdAnnual International Embryo Transfer Society. 7-9 Jan., Kyoto, Japan. Reprod. Fertil. Dev.

[11] Anel L, Alvarez M, Anel E, Martinez-Pastor F, Martinez F, Chamorro C, de Paz P (2011). Evaluation of three different extenders for use in emergency salvaging of epididymal spermatozoa from a Cantabric brown bear. Reprod. Domest. Anim.

[12] Blash S, Melican D, Gavin W (2000). Cryopreservation of epididymal sperm obtainedat necropsy from goats. Theriogenology.

[13] Kundu C.N, Chakraborty J, Dutta P, Bhattacharyya D, Ghosh A, Majumder G.C (2000). Development of a simple sperm cryopreservation model using a chemically defined medium and goat cauda epididymal spermatozoa. Cryobiology.

[14] Tamayo-Canul J, Alvareza M, López-Uruena E, Nicolas M, Martinez-Pastora F, Anel E, Anel L, de Paz P (2011). Undiluted or extended storage of ram epididymal spermatozoa as alternatives to refrigerating the whole epididymes. Anim. Reprod. Sci.

[15] Fernández-Santos M.R, Soler A.J, Ramón M, Ros-Santaella J.L, Maroto-Morales A, García-Álvarez O, Bisbal A, Garde J.J, Coloma M.A, Santiago-Moreno J (2011). Effect of post-mortem time on post-thaw characteristics of Spanish ibex (Capra pyrenaica) spermatozoa. Anim. Reprod. Sci.

[16] Guérin Y, Locatelli Y, Comizolli P, Mauget R, Mermillod P, Legendre X, Gatti J.L, Dacheux J.L (2003). Conservation et utilisation du sperme épididymaire d’ovins et de cervidés en insémination artificielle et fécondation *in vitro*. Les Actes du BRG.

[17] Hishinuma M, Sekine J (2003). Evaluation of membrane integrity of canine epididymal spermatozoa by short hypoosmotic swelling test with ultrapure water. J. Vet. Med. Sci.

[18] Martinez-Pastor F, Guerra C, Kaabi M, Diaz A.R, Anel E, Herraez P, de Paz P, Anel L (2005). Decay of sperm obtained from epididymes of wild ruminants depending on postmortem time. Theriogenology.

[19] Varisli O, Uguz C, Agca C, Agca Y (2009). Motility and acrosomal integrity comparisons between electro-ejaculated and epididymal ram sperm after exposure to a range of anisosmotic solutions, cryoprotective agents and low temperatures. Anim. Reprod. Sci.

[20] Barth A, Oko R (1989). Abnormal Morphology of Bovine Spermatozoa.

[21] Eddy E.M, Neill J.D (2006). Spermatozoon. Knobil and Neill’s Physiology of Reproduction.

[22] Robaire B, Hinton B.T, Orgebin-Crist MC, Neill J.D (2006). The epididymis. Knobil and Neill’s Physiology of Reproduction.

[23] Martins C.F, Driessen K, Melo Costa P, Carvalho-Neto J.O, de Sousa R.V, Rumpf R, Dode M.N (2009). Recovery, cryopreservation and fertilization potential of bovine spermatozoa obtained from epididymides stored at 5 C by different periods of time. Anim. Reprod. Sci.

[24] Lone F.A, Islam R, Khan M.Z, Sofi K.A (2011). Effect of transportation temperature on the quality of cauda epididymal spermatozoa of ram. Anim. Reprod. Sci.

[25] Mir S.S, Lone F.A, Khan M.Z, Malik A.A, Islam R, Sofi K.A (2012). Effect of cold storage period on the quality of ram cauda epididymal spermatozoa recovered postmortem. Turk. J. Vet. Anim. Sci.

[26] Rath D, Niemann H (1997). *In vitro* fertilization of porcine oocytes with fresh and frozen-thawed ejaculated or frozenthawed epididymal semen obtained from identical boars. Theriogenology.

[27] Rickard J.P, Pini T, Soleilhavoup C, Cognié J, Bathgate R, Lynch GW, Evans G, Maxwell W.M, Druart X, de Graaf S (2014). Seminal plasma aids the survival and cervical transit of epididymal ram spermatozoa. Reproduction.

[28] Hishinuma M, Suzuki K, Sekine J (2003). Recovery and cryopreservation of sika deer (Cervusnippon) spermatozoa from epididymides stored at 48C. Theriogenology.

